# Fast Growing Plantations for Wood Production – Integration of Ecological Effects and Economic Perspectives

**DOI:** 10.3389/fbioe.2015.00072

**Published:** 2015-06-09

**Authors:** Michael Bredemeier, Gerald Busch, Linda Hartmann, Martin Jansen, Falk Richter, Norbert P. Lamersdorf

**Affiliations:** ^1^Forest Ecosystems Research Section, Center for Biodiversity and Sustainable Land Use (CBL), University of Göttingen, Göttingen, Germany; ^2^BALSA Service for Applied Landscape Ecology and Scenario Analysis, Göttingen, Germany

**Keywords:** short rotation coppice, energy wood plantations, water budget, nitrogen leaching, integrated landscape assessment

## Abstract

Biomass crops are perceived as a feasible means to substitute sizeable amounts of fossil fuel in the future. A prospect of CO_2_ reduction (resp. CO_2_ neutrality) is credited to biomass fuels, and thus a potential contribution to mitigate climate change. Short rotation coppices (SRCs) with fast growing poplar and willow trees are an option for producing high yields of woody biomass, which is suitable for both energetic and material use. One negative effect that comes along with the establishment of SRC may be a decrease in groundwater recharge, because high rates of transpiration and interception are anticipated. Therefore, it is important to measure, analyze, and model the effects of SRC-planting on landscape water budgets. To analyze the effects on the water budget, a poplar SRC plot was studied by measuring hydrological parameters to be used in the hydrological model WaSim. Results reveal very low or even missing ground water recharge for SRC compared to agricultural land use or grassland, especially succeeding dry years. However, this strong effect on plot level is moderated on the larger spatial scale of catchment level, for which the modeling was also performed. In addition to water, nutrient fluxes and budgets were studied. Nitrogen is still a crucial issue in today’s agriculture. Intensive fertilization or increased applications of manure from concentrated livestock breeding are often leading to high loads of nitrate leaching, or enhanced N_2_O emissions to the atmosphere on arable crop fields. SRC or agroforestry systems on former crop land may offer an option to decrease such N losses, while simultaneously producing woody biomass. This is mainly due to the generally smaller N requirements of woody vegetation, which usually entail no need for any fertilization. The trees supply deep and permanent rooting systems, which can be regarded as a “safety net” to prevent nutrient leaching. Thus, SRC altogether can help to diminish N eutrophication. It is important to offer viable and attractive economic perspectives to farmers and other land managers besides of the potential ecological benefits of SRCs. For this reason, an integrated tool for scenario analysis was developed within the BEST project (“BEAST – Bio-Energy Allocation and Scenario Tool”). It combines ecological assessments with calculations of economic revenue as a basis for a participative regional dialog on sustainable land use and climate protection goals. Results show a substantial capacity for providing renewable energy from economically competitive arable SRC sites while generating ecological synergies.

## Introduction

Since fossil fuels are finite and may become exploited to a critical extent in the near future, there are strong efforts to substitute them as much as possible with renewable energies, and as fast as this could be achieved. Among the diverse renewables, energy from biomass plays an important role. Between the various biomass energy carriers, which are available, wood plays an important part, particularly in thermal conversion. Energy wood can be gained from different sources. Classical forestry has a long tradition, but additional options and strategies to harvest enough energy wood for meeting an increasing demand are needed. The most important option are new energy wood plantations with fast growing woody species and very short rotation periods compared to “classical” forestry. These “short rotation coppices (SRC)” are the focus of our present study, as they may have significant effects on habitat provision, biodiversity, hydrology, and biogeochemical cycling in cultural landscapes. Interdisciplinary investigations of SRC effects were facilitated by the joint integrated project BEST (“Bioenergie-Regionen stärken” – Boosting Bioenergy Regions), which ran from 2010 to 2014 and was funded by the German Ministry of Education and Research (BMBF).

Short rotation coppice does not only provide woody biomass, but frequently additional ecosystem services. If SRC should come to cover larger parts of our landscapes in the future, it is important to know how they will limit or augment ecosystem services in comparison to other land use systems. One known shortcoming of SRC are possible negative effects on groundwater recharge (GWR), because higher rates of transpiration and interception evaporation may be expected compared to annual crops (Lasch et al., 2010; Schmidt-Walter and Lamersdorf, 2012). Due to the change in land use and introduction of trees, new factors occur, which influence water fluxes and their interactions at a site. One factor is the all year vegetation cover in combination with a longer growing season in comparison to annual crops (Petzold et al., 2010). As a result, trees are able to develop a deeper root system and therefore exploit additional water and nutrient resources in deeper soil layers. The more intensive root penetration as well as the more extensive management practice are also affecting soil structure and thus hydrological fluxes, resulting in a higher soil infiltration (Wahren et al., 2012) and reduced runoff components. Compared to typical annual crops, tree dominated land use types like SRC have a higher leaf area index (LAI). LAI has a control on transpiration and additionally influences soil evaporation and interception evaporation. Furthermore, the latter is increased by the interception on stem and branch surfaces, especially outside of the vegetation period. The magnitude of these hydrological effects of SRC highly depends on local climate conditions and soil water availability (Dimitriou et al., 2009), determining actual evaporative demand and water supply. Water availability is the main growth limiting factor for poplar and willow plantations (Linderson et al., 2007). Due to these effects, SRC influences the water fluxes at the plantation site, as well as those of the surrounding landscape.

An assessment using hydrological models can help to identify hydrological effects at regional and local scales, e.g., to regional climate and/or to adjacent catchments. The objectives of the hydrological part of this study were to investigate the effects of an enhanced cultivation of SRC on local and regional scale using measurements and the raster-based hydrological model system WaSim. We used our own measurements of plant physiological parameters like LAI, stomata resistance (Rsc), and the length of growing season to calibrate sensitive parameters for hydrological modeling a the site scale. For SRC, such parameters are quite rare in literature, due to the fact that this land use form scarcely came into focus of investigation before onset of the renewable energy discussion during the last years (Surendran Nair et al., 2012).

In a second step of the hydrological study, we transfer our model parameterization to a whole catchment area, in order to investigate the effects of land use change on a regional scale by analyzing model simulations with different fractions of agricultural area converted to SRC.

Hydrological studies of SRC were complemented by biogeochemical investigations. The focus here was on nitrogen, since nitrogen leaching from the soil to aqueous ecosystems and groundwater is a well-known problem in intensive agricultural land use. N fertilization is often causing serious problems with respect to nitrate leaching and N_2_O emissions in today’s conventional agriculture (Pappa et al., 2011; Snyder et al., 2014). The main reasons for such “N-leaking” are enhanced N doses applied to reach economically attractive production targets of crops, and the inability to exactly synchronize N needs for plant growth with the time frames of N applications (Di and Cameron, 2002). Furthermore, calculations of N demands are related to the needs of sufficient plant growth rather than to the capacities of soils to intermediately store excessive loads of N from fertilization or crop residue mineralization. Thus, most N leaching can be expected on rather poor and light soils, where high production targets should be accomplished by high inputs of N fertilizers. In addition, the persistent use of heavy agricultural machinery is often causing serious soil compaction on intensively used crop fields which is, together with a surplus of nitrate, favoring the genesis and emission of N_2_O (Ruser et al., 2006).

With respect to the leaching of N, SRC is known to generally reduce the output of nitrate on former cropland due to the smaller net N demand of woody perennials (Dimitriou et al., 2009; Dimitriou and Aronsson, 2011; Schmidt-Walter and Lamersdorf, 2012). Thus the cultivation of SRC, in the form of size restricted monoculture blocks in-between the existing N intensive crop applications, or as strips in the form of windbreaks (alley cropping systems), riparian buffers, or other agroforestry measures may help to restrict the above mentioned negative impacts of large and N intensive monocultures (Tsonkova et al., 2012). However, the specific implementation of SRC as buffer strips for nitrate leaching would be a fairly new application in central European agricultural land-use. In this context, our main goal was to investigate key hydrological conditions and terms of the N cycling for a newly installed SRC plantation with poplar and willow on former cropland. Finally, we were focusing on setting up first N-budgets for those young SRC plantations to better understand and evaluate their performance under initial growth conditions.

Perennial crops like SRC can effectively reduce the risk of water erosion, since they provide a soil surface cover of living vegetation and plant detritus. Surface runoff is considerably reduced and infiltration is supported due to an extensive rooting system, humus incorporation into the soil, and lower management frequency (e.g., harvest operations; Kort et al., 1998; Scholz et al., 2001; Deumlich et al., 2006). Since soils in the district of Göttingen are very exposed to water erosion (Mosimann et al., 2009), owing to their soil texture characteristics and the hilly landscape, SRC could play a substantial role in erosion prevention.

Ecological effects are an important concern with respect to establishment of new SRC. However, economic questions and considerations are also essential. Farmers will never adopt SRC cultivation if they do not perceive this land use form as an economically sound and promising solution. It is therefore highly important to offer viable and attractive economic perspectives to farmers and other land managers besides of the potential ecological benefits of SRCs. To address this issue, an exemplary scenario study was carried out focusing on two major objectives: (a) to derive the maximum amount of economically competitive primary energy production from SRC that could be generated from arable land in the municipality of Friedland (78 km^2^, southern district of Göttingen), and (b) to analyze whether ecological synergies in terms of erosion protection could be achieved with an economically guided site selection.

“Economically competitive” means that SRC has to generate a positive annual economic return (annuity) compared to the reference crop rotation of wheat–oilseed rape–barley within a 20-year time period 2011–2030 (Forstliche Versuchs- und Forschungsanstalt Baden Württemberg (FVA), 2012).

## Materials and Methods

### Description of study site, catchment, and measurements

Short Rotation Coppice research plantations was established in the Göttingen district, Central Germany. The site considered here is located next to the village Reiffenhausen (51.67°N, 10.65°E, 325 m a.s.l.), and is named “Reiffenhausen” accordingly.

The soil substrate at the Reiffenhausen site is characterized by sedimentary deposits of Triassic Sandstone material, partly mixed with clay stone material, and covered by loess sediments. The soil types are stagnic cambisols (pseudovergleyte Braunerde) and haplic stagnosols (Pseudogley). The soil texture is dominated by a loamy sand or silty clay fraction [for further details see also Hartmann et al. (2014)]. The SRC plantation was established in March 2011 and consists of two pure SRC plots, one planted with the poplar clone “Max 1” (*Populus nigra* × Populus maximowiczii), hereafter called P-SRC, and another one planted with the willow clone “Tordis” [(Salix viminalis × Salix schwerinii) × Salix viminalis)] hereafter called W-SRC.

The P-SRC plot was planted with 0.2 m long poplar cuttings on 0.4 ha area in double row spacing system, comprising alternating inter-row distances of 0.75 and 1.50 m, and a distance of 1.0 m within the rows. The overall planting density was 8890 cuttings ha^−1^. The W-SRC plot has a dimension of 0.6 ha and a planting density of 11,850 cuttings ha^−1^. Willow clone “Tordis” cuttings of 0.2 m length were planted also in double rows with the same inter-row distance as poplar, but the distance within the rows was only 0.75 m.

In 2013, the most intensive measurements and investigations took place. Measured parameters were LAI, stomatal resistance (Rsc), plant height, and diameter at breast height (DBH), both as annual development, rooting depth, as well as start and length of the growing season. Besides these plant characterizing parameters, also local climate and soil physical parameters were recorded. A detailed description of the investigation of these values is given by Richter et al. (2015); information about soil chemistry and biomass information is given by Hartmann et al. (2014). According to the meteorological data provided by the German Weather Service (Deutscher Wetterdienst, DWD), for the station Göttingen (DWD Station-ID: 01691), nearest to the study site, the climate is characterized by an average temperature of 9.1°C (±0.7°C), and a mean annual precipitation sum of 635 mm (±122 mm) for the period 1971–2010.

**Figure 1:**
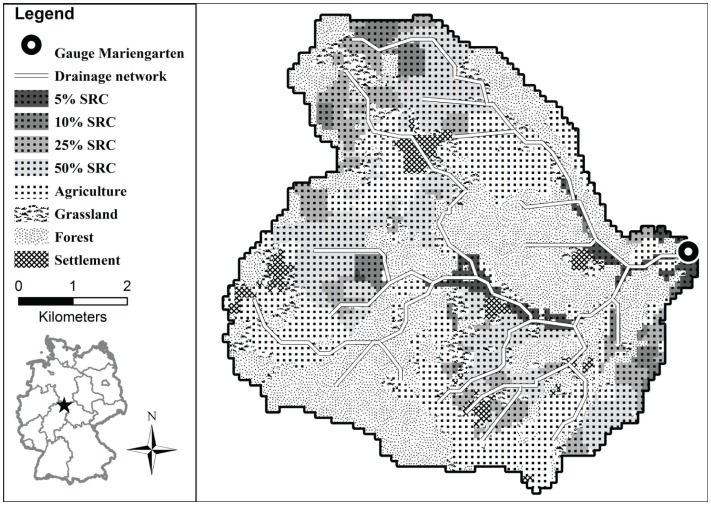
**Raster map of the land use composition in the Dramme catchment**. Percentages of agricultural area changed to SRC are additive, i.e., 10% SRC includes the 5% SRC areas, etc.

For regional scale modeling, the catchment of the Dramme River is used, which is a small stream southeast of Göttingen, flowing into the Leine River. The catchment has an area of 45 km^2^, and elevation is ranging from 198 to 476 m a.s.l. The catchment used here is defined down to the discharge gage Mariengarten (Niedersächsischer Landesbetrieb für Wasserwirtschaft, Küsten- und Naturschutz (NLWKN), 2013a), where runoff data is available. Half of the catchment area is used for agriculture, 36% is covered by forest, where 23% of the total area is deciduous forest. Grassland covers 9% and the remaining area are settlements or water bodies. This landscape characteristic is typical for central Germany (Figure 1). A detailed description is given by Richter et al. (2014).

**Figure 2:**
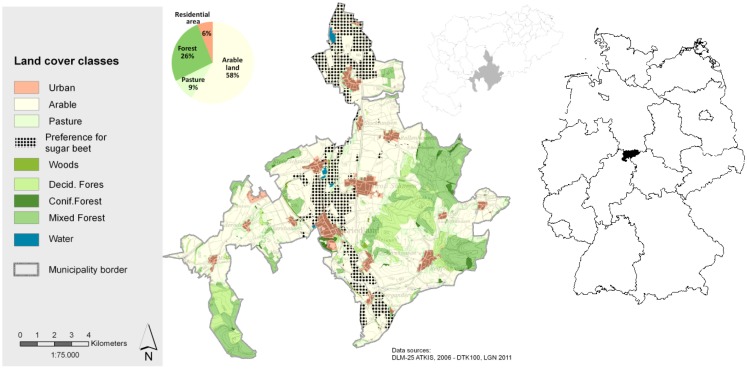
**Landscape mosaic in the municipality of Friedland including preference sites for sugar beet–wheat rotations that are excluded from the scenario assessment**.

A software tool named “BEAST” was developed within the BEST project (“Bioenergy assessment and scenario tool”; Busch and Thiele, 2015). The BEAST scenario application referred to the municipality of Friedland (Figure 2), which is located in the southern parts of the Göttingen district. The municipality is dominated by arable land use, covering 54% of the total area (76 km^2^). Natural growth conditions are suitable for SRC, given an average annual precipitation of around 700 mm, a mean annual temperature of 8.5°C (1981–2010 – derived from DWD 1 km grid information; DWD German Weather Service, 2013), and a majority of medium to high productive soils.

### Hydrological modeling approach

To investigate the effects of an enhanced cultivation of SRC on hydrology, we applied a three-step approach. The first step was the measurement and derivation of model relevant parameters, especially describing the land use form “poplar SRC” on the plot level. The measurements of plant physiological parameters and a sensitivity analysis of how different vegetation parameterizations can influence hydrological model results are presented in Richter et al. (2015).

In the second step, WaSim was applied at the plot scale to Reiffenhausen P-SRC, where measurements were done to calibrate and evaluate the model using independent measurements, i.e., soil moisture quantities in our case. This plot model approach of WaSim was also used to perform long-term simulation to study the effects of climate variability as well as different land use and soil types on model results (Richter et al., 2015). The third step was the investigation of hydrological effects of SRC cultivation on the regional scale, by example of the small catchment area of the river Dramme. Here, the parameterization for poplar SRC, derived on the plot level, is used together with other land use types to investigate local and regional changes of hydrological properties for different scenarios of SRC cultivation. Scenarios were selected for different area fractions converted from agriculture to SRC, i.e., 5, 10, 25, and 50% (Figure 1). The rationale behind taking the conversion steps of 5–25% was to reflect biomass supply goals of existing policy scenarios (Landkreis Göttingen, 2013). For hydrological reasons, i.e., to create a strong hydrological signal, a simulation comprising 50% of converted arable land was carried out. The selection process of the most suitable arable sites to be converted to SRC built on a site-specific comparison (performed with the help of BEAST, see below) between yield levels of annual crops (reference crop rotation of oilseed-rape, wheat, and barley) and a poplar SRC (Max1 clone, 5-year rotation). Yield levels were derived from a statistical modeling approach applied to the Göttingen district, which in turn built on field experiments in Lower Saxony and Thuringia (Busch and Thiele, 2015). By running a MIN–MAX optimization routine, sites were selected that ideally show both, comparatively low average agricultural crop yields and at the same time sufficiently high average annual SRC yield levels, respectively.

### N budget measurements and calculations

To estimate the initial N-budget as well as nitrogen use efficiencies of the growing SRC plantations, the net-N-mineralization rates (NNM), plus the atmospheric deposition (AD) were taken as input terms. The net N uptake by trees (NTU), i.e., the amount of N which would leave the system through harvesting measures (including bark), as well as the current seepage water output of nitrate (NSWO) were taken as proxies for the output term. The rates of N_2_O emissions, determined by Walter et al. (2012) through a parallel running project, were only recorded on the P-SRC plot but were also taken for the budget calculations of the W-SRC plot. For NNM, the buried bag method according to Eno (1960) and Gasche et al. (2002) was applied. Given rates are means of eight sampling dates between March and October 2012. During each 6-week incubation period, air-permeable and watertight PE-bags were buried in 15 cm soil depth.

NNM in [mg kg^−1^ d^−1^] was calculated as follows:

(1) $$\text{NNM=}\frac{\left(\text{Ae+Ne}\right)\text{-}\left(\text{Ai+Ni}\right)}{t}$$

where Ae, respectively Ne, is the amount of K_2_SO_4_ (0.5 mol L^−1^) extractable ammonium (Ae) and nitrate (Ne) at the end of the incubation period, Ai and Ni are respective amounts at the beginning of the incubation period, and *t* denotes the applied incubation period in days [d]. All samples were transported and stored in a cold chain, and were immediately extracted in the lab after the field sampling. ${\text{NH}}_{4}^{+}$ and ${\text{NO}}_{3}^{-}$ were determined by using continuous flow injection calorimetry (Cenco/Skalar Instruments, Breda, the Netherlands).

Atmospheric deposition was measured bi-weekly with four replicated bulk samplers per plot between November 2011 and October 2013. NTU was estimated by determining total N concentrations in 15 shredded trunks per plot. After drying at 60°C and grinding the plant material to a fine powder, total N was analyzed with an automated CHN-O-analyzer (VarioEL, Elementar, Germany). Subsamples were also dried at 105°C to determine the remaining water content, and reported results are related to the absolute dry weight. For the estimation of the above-ground biomass production, the DBH (1.3 m) was determined for each plot at randomly selected trees (*n* = 240). Those measurements were applied after the second growth period, i.e., in winter 2012/13. Fifteen trees per plot were harvested at 10 cm above soil level and the fresh-weight was determined *in situ*. After dry-weighing to constant mass (105°C), allometric equations given by Röhle and Skibbe (2012) were used to estimate the dry mass of trees. Finally, these estimates were scaled up with the number of survived trees to calculate dry matter (DM) yields per hectare [for further details on biomass estimates, see also Hartmann et al. (2014)]. The NSWO was determined by (i) measuring monthly nitrate concentrations in the soil solution from four replicated suction lysimeter cups per plot in 60 cm soil depth between December 2011 and November 2012, and (ii) multiplying monthly mean nitrate concentrations with respective soil water fluxes, gained from the modeling activities described in the section above. N_2_O emissions were recorded on the P-SRC plot between October 2011 and October 2013 (Walter et al., 2012). Furthermore, the N-uptake efficiencies (NUP-E) as well as N-utilization efficiencies (NUT-E) were calculated according to Moll et al. (1982). NUP-E is the rate of tree N uptake per sum of N inputs [NTU/(NNM + AD)], whereas NUT-E is the rate of biomass produced per unit N uptake by trees (DM/NTU). The respective units for those efficiencies are [kg kg^−1^] but may be also given as [%] for NUP-E.

### Model scenario setting for a 20-year period (2011–2030) case study simulation

#### Site Selection for the Scenario Application

Arable sites that are preferable for a wheat–wheat-sugar beet rotation were identified and excluded from the assessment procedure since sugar beet production will still be subsidized within the next years and the economic return out-competes other crops, including SRC. Thus, by taking soil quality and slope as parameters for site selection, around 883 ha (21% of 4265 ha of arable land) were identified as preference sites for sugar beet rotations in the study area (Figure 2). For the remaining 3381 ha of the arable land, the scenario setting (Table 1) was applied in order to determine both, economic feasibility and options for erosion protection. Spatial modeling and economic calculation took place on the arable site level (i.e., 1109 arable fields).

**Table 1:** **Scenario setting for the BEAST assessment in the municipality of Friedland**.

Item	Description/rule	Expected value	RMSE
**Production criteria**
Yield level crops/yield variation	Average yield level (2011) for reference crops (model results) in decitons (dt) in the study area	84 (wheat)	4.1 dt (W)
		80 (barley)	4, 4 (B)
		41 (oilseed rape)	2, 1(OR)
Yield increase crops	Trend analysis (1981–2011) of annual yield increase for reference crops in the Göttingen district	0.7% (wheat)	–
		0.5% (barley)	
		0.5% (oilseed rape)	
Yield level SRC	Mean annual increment (MAI) over a 20-year period (5-year rotation) for MAX-1 poplar SRC with 7, 000 cuttings in the study area	13.5 oven dry tons (odt)	–
**Economic criteria**
Commodity prices/price changes	Input price level 2011 (net) in € per dt	20, 5 (wheat)	3, 9(W)
		18, 8 (barley)	5,1 (B)
		44, 2 (oilseed rape)	5, 3 (OR)
	Input price level 2011 (net) in € per odt (oven dry ton) of wood chips	91	4
Crop production costs (€)	Yield-specific functions for labor costs, variable costs, fix machinery costs (2011 level)	5, 1133× + 752, 72 (W)	–
		3, 5015× + 774,93 (B)	
		3, 2467× + 1014,4 (OR)	
		(*x* = annual yield)	
SRC production costs (€)	Establishment	2650	–
	Harvest operation and site management	12×+600	–
		(*x* = yield per rotation period)	
	Re-conversion	1950	–
**Ecological state criterion**
Disposition to water erosion	Sites with a potential soil erosion >30t ha^−1^ a^−1^ according to EU Cross Compliance regulation
**Spatial restrictions**
Restricted areas	FFH, nature sanctuaries
Buffer zones	Distance to humid biotopes >200 m
**Objectives**
Economic return	Annuity difference for SRC >0 € compared to annual reference crops
Ecological synergies	Potential synergies of erosion protection, renewable energy production, and economic return

Further, for reasons of government payments (“NAWARO-Bonus”), only arable sites with an area >0.3 ha and <10 ha were considered. To avoid potentially negative effects from SRC on humid biotopes, protection buffer zones of 200 m were applied to these biotopes. For reasons of nature conservation, nature conservation areas and FFH (Flora-Fauna-Habitat) zones were excluded from SRC allocation. Information on humid biotopes and target areas for nature conservation were taken from datasets provided by Niedersächsischer Landesbetrieb für Wasserwirtschaft, Küsten- und Naturschutz (NLWKN) (2013b).

#### Cost and Price Calculation

The information used to calculate yield-related costs for the annual reference crops and for a poplar SRC in a 5-year rotation originated from the Niedersachsen Chamber of Agriculture (LWK, 2002–2013), the German Farmers’ Association (Wagner et al., 2012), and various other sources [e.g., Nahm et al. (2012), Strohm et al. (2012), Forstliche Versuchs- und Forschungsanstalt Baden Württemberg (FVA) (2012), Kröber et al. (2010), and Landesanstalt für Entwicklung der Landwirtschaft und der ländlichen Räume (LEL) (2010)].

For the cost calculation, a distinction was made between variable costs, labor costs, and fixed costs for machinery. Fixed costs for arable crop production were considered since the cost calculation available for SRC (planting, harvesting, re-conversion) was based on subcontracting, and, thus, included fixed costs for machinery. Overhead costs, rents, and EU single farm payments were not considered as these parameters do not differ between SRC and annual crops. Commodity prices were derived from regional and national statistics (C.A.R.M.E.N. e.V, 2014; EUWID, 2014; Federal Statistical Office – Statistisches Bundesamt, 1980–2001; LWK, 2002–2013). These prices were calculated as net prices without VAT (Table 1).

As drying, storage, and transportation to the farm were considered when calculating SRC-related costs, the prices were adjusted by subtracting 15 € per odt of wood chips (Wagner et al., 2012) from C.A.R.M.E.N price statistics. The net price for woodchips from SRC was further adjusted to include drying losses (−20%), in accordance to Bärwolff and Hering (2012) and Kuratorium für Technik und Bauwesen in der Landwirtschaft (KTBL) (2012), respectively. Net commodity prices for wheat, barley, and oilseed rape were directly taken from Federal State statistics of Lower Saxony (LWK, 2002–2013). For both price and cost calculation, 2011 numbers were taken as reference (Table 1).

#### Yield Modeling and Potential Primary Energy Supply from SRC

The yield data for the annual reference crops (i.e., wheat, oilseed rape, and barley) reflect modeling results of average decadal yields, whereas the SRC yield data (poplar SRC, 5-year rotation, 7000 saplings) refer to the simulated mean annual increment of woody biomass per rotation period. Modeling results are provided for four rotation periods (Busch and Thiele, 2015). Crop yield data for the annual reference crops (i.e., wheat, oilseed rape, and barley) were generated by a statistical yield model based on climate and soil parameters as well as yield information from 52 sites all over Lower Saxony, Germany (LWK, 2002–2013). Model results were calibrated and validated for the Göttingen district (Busch and Thiele, 2015).

Mean annual increment of the MAX-I poplar SRC was simulated according to a modified approach published by Petzold et al. (2014). The approach is based on the relation between mean annual increment of biomass and available soil water capacity, which provides a function of potential annual biomass production for a specific plantation age. Busch and Thiele (2015) adapted the approach to three long-term data sets of yield levels (Max-I poplar) from Thuringian sites (Thüringer Landesanstalt für Landwirtschaft (TLL) (2010, 2012)). They introduced an age-specific modifier to the yield function that allows to model yields for various ages and rotation periods of SRC.

Short Rotation Coppice yields were transformed to numbers of primary energy supply by using a conversion factor of 4.95 MWh per oven dry ton of biomass yield according to Fachagentur Nachwachsende Rohstoffe (FNR) (2012).

#### Dynamic Investment Calculation over a 20-Year Period (2011–2030)

An economic comparison between perennial and annual cropping systems needs a dynamic investment cost calculation because timing of cash flows in these systems is different. As a result of this approach, annuities for both systems (reference crop rotation and poplar SRC) were calculated and related. Annuities in turn describe the annual constant cash flows over the investment period.

To determine economic return of both, the reference crop rotation and the SRC system, some boundary conditions were set: (a) reference prices and costs are the numbers of 2011, (b) to allow for a better comparison, no price and cost increase over the simulation period was assumed, (c) yield increase of annual crops was considered according to trend analysis (Busch and Thiele, 2015 – see Table 1), (d) price and yield fluctuations were considered according to recent trends (2002–2011) via Monte Carlo simulation (MC).

Taking the 2011 yield- and price-levels as input values for the MC, the RMSE (root mean square errors) of the underlying yield and price models for the period 2002–2011 were used to determine plausible variations of change (see economic and production criteria in Table 1). The RMSE were interpreted as equivalents to the SDs of Gaussian distributions. That way MC simulation randomly changed the expected input values (i.e., yields, prices) within the range of RMSE and iterated 10,000 times to generate statistically valid output. The results from the MC simulation provided a frequency distribution and, thus, allowed for deriving the probability of a positive economic return from SRC compared to the reference crop rotation.

Commodity prices for the annual reference crops as well as the wood chip prices were highly correlated within the 2002–2011 decade. Thus, for each iteration, one Gaussian random number of price change within the MC simulation was applied for all commodities, but scaled according to the crop-specific RMSE (Table 1).

#### Identification of Arable Sites which are Prone to Water Erosion

For the ecological synergy analysis, only arable sites were considered that show a potential water erosion rate higher than 30 t ha^−1^ a^−1^ since this is the threshold where Farmers have to take protective measures according to EU Cross Compliance (EU, 2009) regulation.

Disposition to water erosion was derived by building on input information from LBEG (Müller and Waldeck, 2011). As a federal state agency, LBEG provides reference methodologies for soil assessments. The assessment on disposition to water-induced soil erosion is based on the German adaptation of the Universal soil loss equation (USLE), and refers to soil information of the German Soil Survey (1:5.000) and a digital elevation model with 12.5 m grid. Details can be found in Schäfer et al. (2010).

## Results

### Hydrological field measurements and plot level modeling

**Figure 3:**
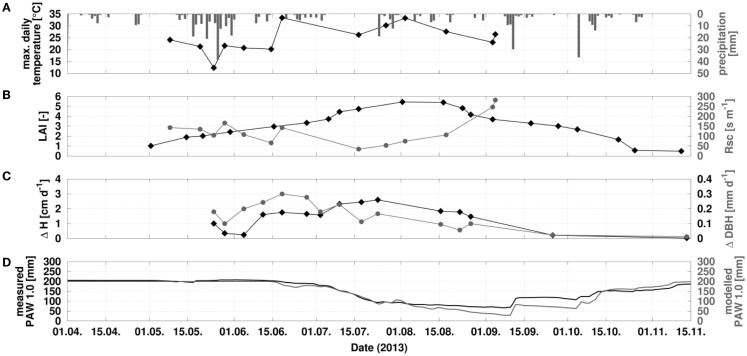
**Time series of daily max. temperature, precipitation, leaf area index, stomatal resistance, growth parameters, and soil water changes of a poplar SCR in Reiffenhausen (Lower Saxony) in 2013**. **(A)** Daily max. temperature and precipitation; **(B)** measured course of leaf area index and stomatal resistance; **(C)** measured growth rate of tree height (ΔH) and diameter at breast height (ΔDBH); **(D)** measured and modeled course of plant available water, calculated until 1 m soil depth (PAW1.0). Black drawings with diamonds belong to the right axis, gray ones with circles to left axis.

Figure 3A shows precipitation volumes in Reiffenhausen from April to November 2013. The annual precipitation in 2013 amounted to 640 mm (DWD station Göttingen), which is close to the long-term mean of 635 mm (period 1971–2010). The measured plant available water down to 1 m soil depth (PAW1.0) reflects the sequence of precipitation events as illustrated in Figure 3D. After intense rain events in May 2013, only minor or short events occurred which were not able to prevent the soil from drying. Starting from August, drought stress occurred, which is also the reason for the increase in Rsc (stomatal resistance), illustrated in Figure 3B. Besides drought stress, also air temperature is affecting Rsc, which is illustrated by comparing the Rsc measurements with the daily maximum temperature (Figure 3A). At the beginning of September 2013, the wilting point was already reached, affecting biomass growth as illustrated in Figure 3C, as well as changes in plant height and diameter at breast height. Figure 3D compares PAW1.0 as measured and modeled using a parameterization of poplar SRC based on measurements, such as the annual course of LAI (Figure 3B). As an integrated soil hydrological value, PAW1.0 indicates a good model agreement, resulting in a Nash–Sutcliffe model efficiency coefficient of 0.9.

**Figure 4:**
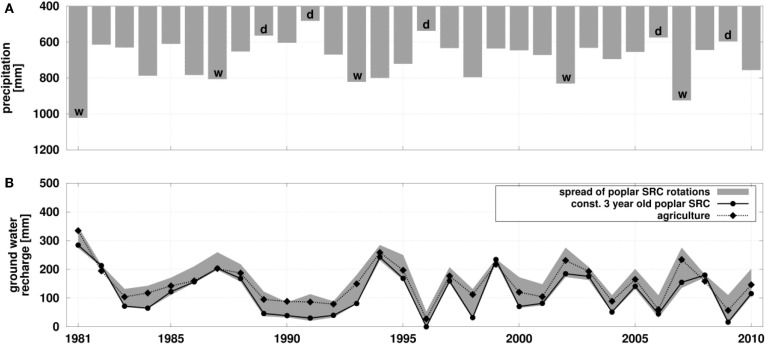
**Comparison of plot model results for the two land use types agriculture and poplar SRC – with and without rotation**. **(A)** Sums of precipitation for hydrological years (Oct–Nov), “d” marks the 5 driest years and “w” the 5 wettest years in the period 1981–2010; **(B)** sums of ground water recharge for hydrological years for agriculture and poplar SRC, whereas poplar SRC is kept constant in time (no rotation, 3-year-old) and the spread over simulations using a 5-year rotation period, starting in each of these 5 years.

Figure 4 shows results of long term simulations performed on the plot level for Reiffenhausen using this calibrated and parameterized model setup by forcing the model with climate data from the DWD station Göttingen. Figure 4A shows the annual precipitation. Lines in Figure 4B compare the modeled GWR of agriculture and poplar SRC, with no rotation considered, i.e., permanently parameterized as a 3-year-old poplar SRC. The GWR for poplar SRC is lower compared to agriculture. There is an increase in years with reduced or even missing GWR for poplar SRC, especially in succeeding dry years. The gray shading in Figure 4B represents the spread of five simulations reflecting the growth of poplar under consideration of a 5 years rotation period. Each simulation starts with one of the 5 rotation years to cover all possible combinations of rotation years and climate forcing present in this period.

Figure 4B shows a wide spread of GWR for these five simulations, where especially the first and second year of rotation are characterized by lower evapotranspiration (ETR) and therefore resulting in GWR rates comparable to or only slightly lower than for agriculture (Richter et al., 2014). Due to the consideration of the 5-year rotation period, GWR rates can be higher or lower, compared to the SRC simulation with no rotation depending on the overlap of dry or wet years with the distinct rotations years.

On average, over all five rotation simulations ETR is lower, and GWR is higher than in the simulation with no rotation. A detailed analysis of the five rotation simulations shows that the highest GWR occurs not in the first rotation year, when the poplar SRC is small in height and LAI and therefore ETR is less, but in the second rotation year. The first year of rotation is needed to refill the soil water storage, which is exploited during the 3rd, 4th, and 5th year of rotation due to the high ETR. The soil water storage of the silty soil in Reiffenhausen is high; therefore, the refilling takes some time and only after that GWR can occur, i.e., mostly in the second rotation year. Only when precipitation is high, GWR is largest in the first year of rotation, for instance in 1981.

### Regional scale hydrological modeling

**Figure 5:**
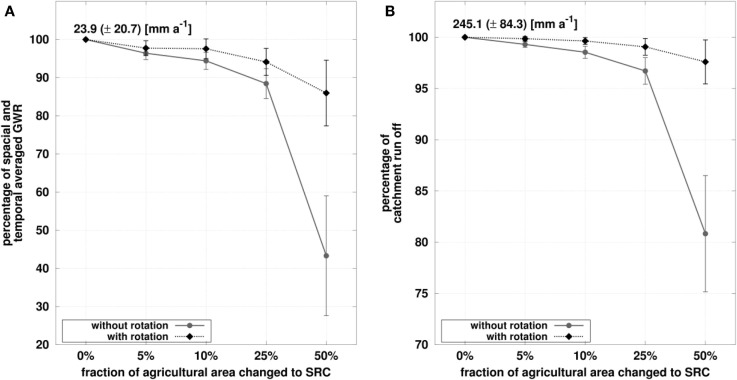
**Change of ground water recharge (GWR, spatial mean over catchment area) and catchment run off relative to reference simulation (0% SRC)**. Mean values and SDs of relative changes are calculated over all simulated years, e.g., 30 for the simulations without rotation and 5 × 30 for the simulations with rotation. **(A)** Percentage of spatial and temporal averages ground water recharge (GWR). **(B)** Percentage of catchment runoff.

Figure 5 shows the changes of averaged GWR and catchment runoff for the simulations with different fractions of agricultural area changed to SRC relative to the reference run, i.e., no SRC. The changes of spatially and temporally averaged GWR and catchment runoff are smaller for the simulations considering the SRC rotation. The effects for the 5 and 10% land use change simulations are moderate, <10% reduction of GWR, and <3% for the runoff, regardless of the rotation consideration. Starting from a 25% land use change, the effects of considering SRC rotations become larger. Replacing half of the agricultural area with poplar SRC reduces the GWR by 57% (± 16%) without rotation and by 14% (± 9%) with rotation. Catchment runoff is reduced by 19% (± 6%) without rotation and by 2% (± 2%) with rotation. Besides these averaged and aggregated signals, large local changes of GWR are present at specific locations in the catchment. These local reductions of GWR are comparable to the plot model results shown in the former section; however, they are of different magnitude due to different soil physical properties in the catchment, compared to the plot Reiffenhausen (Richter et al., 2014).

### Nitrogen budget

Total input of N, defined here as the mean rate of NNM plus the input rates of AD for the observation period, summed up to almost 30 kg ha^−1^ a^−1^ N for P-SRC and 40 kg N ha^−1^ a^−1^ for W-SRC (Table 2). The higher N input for W-SRC is obviously caused by enhanced rates of NNM, but the differences are not significant due to high spatial and temporal variability. NTU is obviously lower for W-SRC compared to P-SRC, even if biomass production (DM) was almost the same on both plots (P-SRC 1.0 t, W-SRC 1.1 DM ha^−1^ a^−1^), measured after the second growth period, i.e., in winter 2012/13 [for more details, see Hartmann et al. (2014)]. On the other hand, the output of N, primarily driven by nitrate leaching at both plots and obviously less important for N_2_O, was significantly higher under willow, compared to poplar. Considering the time pattern of the nitrate leaching under W-SRC over the entire observation period (December 2011–November 2012; not shown), it becomes obvious that those enhanced mean leaching rates under W-SRC were driven by high initial NO_3_-N-concentration in soil solution (spring 2012 with max. of 33 ± 8.7 SD mg NO_3_-N L^−1^). But already since August 2012, nitrate concentrations under willow strongly decreased to the level of the P-SRC plot, i.e., to a range between 0.1 and 2 mg NO_3_-N L^−1^. Enhanced initial nitrate concentrations under Poplar occurred only in May 2012 (7.3 ± SD 8.7 mg NO3-N L^−1^) but remained almost constantly under the detection limit toward the end of the observation period.

**Table 2:** **Mean (±SD) initial N-budget and N-use efficiency estimates for SRC in Reiffenhausen comprising poplar (P-SRC) and willow (W-SRC) plantations**.

Plot	P-SRC	W-SRC
	
Input	[kg ha^−1^ yr^−1^]	
Net-N-Mineralization (NNM)	18.6 (13.3)	28.1 (25.5)
Atmospheric deposition (AD)	11.0 (0.4)	11.0 (0.4)
Σ Input	**29.6**	**39.1**
**Output**		
Net uptake by trees (NTU)	7.4 (0.9)	5.5 (0.9)
NO_3_ seepage water output (NSWO)	2.5	22.3
N_2_O emission	≤2	≤2
**Σ** Output	**11.9**	**29.8**
**N-Budget (Input–Output)**	**17.7**	**9.3**

	**[kg kg^−1^]**	

N-uptake efficiency [NTU/NNM + AD]	0.25	0.14
N-utilization efficiency [DM/NTU]	135	198

### Aspects of SRC allocation according to the case study scenario

#### Economic Competitiveness

**Figure 6:**
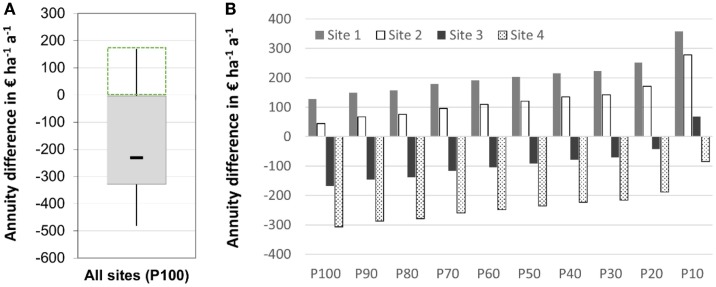
**Minimum annuity difference (i.e., the annuity difference that was simulated with a 100% probability – P100) between poplar SRC and a reference crop rotation (wheat-oilseed rape-barley) for (A) all arable sites being subject to the reference crop rotation – dashed green box indicates the sites with a positive annuity difference as shown in more detail in Figure 7A, and (B) probability related annuity differences for exemplary sites to illustrate “probability profiles” of economic return (P10–P100 illustrate the percentiles of probability)**.

Monte Carlo simulation results (10,000 runs) revealed that the majority of arable sites in the case study area are not capable to provide a positive economic return for SRC under all circumstances (Figure 6A). Numbers presented in Figure 6A reflect the 100% probability situation for all scenario runs, i.e., the minimum annuity difference of SRC that could be generated on each particular arable site. Thus, in 81% of all cases simulated in this scenario, the minimum annuity difference of SRC is lower than the reference crop rotation of wheat, oilseed rape, and barley. In 19% of the scenario cases, however, MC shows that SRC out-competes the reference crop rotation with a 100% probability. Here, the minimum annuity difference which a farmer could count on ranges between 0 and 180 € (Figure 6A). Note, however, that even sites with a negative minimum annuity difference could reveal positive annuity differences – but with lower probabilities (Figure 6B). Since MC was carried out for each arable site in the study area, it is possible to depict “probability profiles” of annuity differences (Figure 6B) and to support farmer’s decision making.

**Figure 7:**
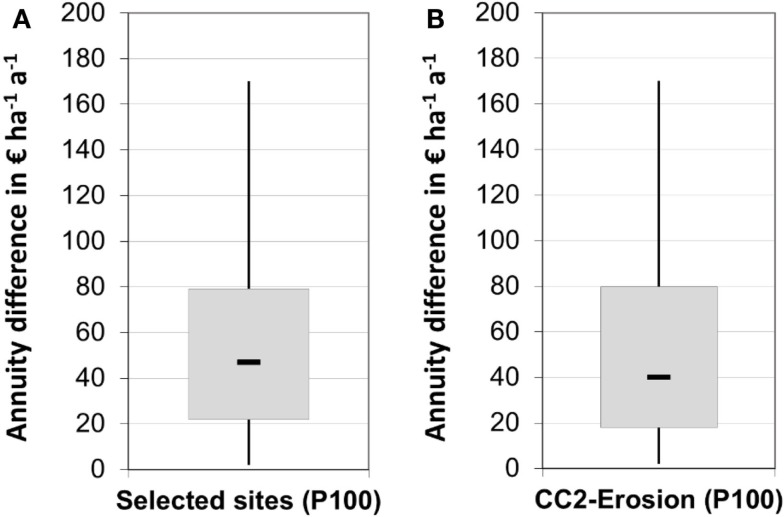
**Annuity difference between poplar SRC and a reference crop rotation (wheat-oilseed rape-barley) for (A) selected economically competitive sites with positive economic return in all scenario cases (P100), and (B) ecological synergy of erosion protection on these economically competitive sites**.

If farmers want to avoid the risk of a negative annuity difference under all circumstances, they have to stick to these 19% of arable sites shown in Figure 6A. Figure 7A depicts the value distribution of these particular sites with a median value of 47 €, and 75% of the numbers ranging from 30 to 75 €. In terms of area, this selection accounts for 541 ha (16%) of the arable land in the case study region.

Again, farmers could use the probabilities of annuity differences delivered by the MC simulation (Figure 6B) to get a more detailed impression of site-specific economic SRC potential, and e.g., to weigh minimum annuity differences (P100) against numbers of median probability (P50) or the complete “probability profile.”

#### Erosion Protection

Taking the 541 ha with a minimum annuity difference >0 € as the spatial reference, intersection with erosion mapping revealed that about 20% (110 ha) of these areas are prone to a very high risk of soil erosion (CC-2 level) and need protective measures according to Cross Compliance regulation. Figure 7B shows that SRC implementation and erosion protection on these sites could be combined without hampering economic return. The median value only slightly decreases to 42 € and the box-range of minimum annuity difference values widens a bit compared to the spatial reference situation (Figure 7A). Since erosion protection is an environmental protection goal that is subsidized by government payments, minimum economic return would increase, which in turn could be an additional incentive for a SRC implementation on these particular sites.

#### Primary Energy Supply

Taking into account the 541 ha with a 100% probability of positive economic return, the average annual biomass production within a 20 year time frame is 9635 odt of wood which, in turn, is equivalent to 48.2 GWh of annual primary energy production. These sites show an average yield level of 17.4 odt a^−1^, which corresponds to an energy density of around 85 MWh ha^−1^ a^−1^, and is, thus, equivalent to maize production on a 500 dt annual yield level (Kuratorium für Technik und Bauwesen in der Landwirtschaft (KTBL), 2012). If the spatial reference is restricted to sites which are subject to high risk of soil erosion, the primary energy supply diminishes to 9.5 GWh a^−1^, while the average yield level only slightly declines to 17.2 odt a^−1^.

#### Potential Impact on Humid-Sensitive Habitats

**Figure 8:**
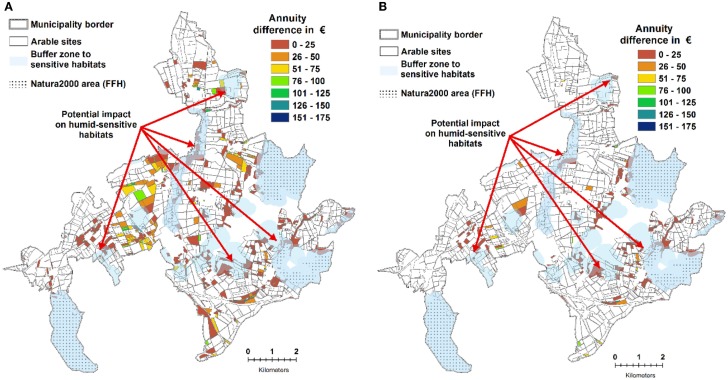
**(A)** Economically competitive SRC sites, **(B)** economically competitive SRC sites that provide cross compliance-relevant erosion protection. **(A)** additionally depicts layers addressing potential restrictions for SRC allocation.

Locally, SRC can have a negative impact on habitats that rely on high groundwater level or soil interflow from neighboring areas. Around 58 ha of the potential 541 ha of SRC sites were identified within a 200 m buffer radius of riparian FFH areas (Figure 8A), and another 38 ha could have an impact on neighboring humid-sensitive habitats. Thus, a potentially negative effect of SRC on surrounding habitats has to be carefully considered. However, 25 ha of these particular sites show a very high disposition to soil erosion which needs to be taken into consideration as well (Figure 8B).

## Discussion and Conclusion

More cultivation of SRC changes water fluxes on both the plot and the landscape scale. On the plot scale, it was shown that the water consumption of SRC is all together higher in comparison to annual crops. Locally, this can result in a significant reduction up to the ceasing of GWR. So, the high water demand of poplar SRC could be confirmed by the measurements and simulations on the plot scale, as well as the link between sufficient water availability and optimal biomass growth.

The higher water demand results from the higher evapotranspiration of SRC compared to annual crops. This is especially due to a longer vegetation period, a deeper and more developed rooting system, as well as higher interception evaporation, because of larger LAI values. However, these effects differ in a wide range depending on the site conditions and the SRC management (Stone et al., 2010). The latter has the potential to shift site-specific changes in water fluxes into the desired direction. Reduced GWR leads to a lower mass transfer from soil to ground water and can reduce nitrate leaching. But the higher water demand of SRC also involves the risk of water shortage, which could be critical in areas where a certain water supply is needed (Le et al., 2011), e.g., drinking water reservoirs or ecosystems dependent on a minimum ecological runoff. However, to observe a significant reduction signal on the catchment scale, (averaged GWR and run off) more than 25% of the catchment would have to be converted from agriculture into SRC. Nevertheless, effects of SRC cultivation on site water fluxes can be locally significant, especially for sensitive ecosystems and in dry years.

With respect to biogeochemical effects, the conversion of conventional crop fields into SRC plantations may also cause significant changes. One of the most prominent and initial effects is the end of N input by fertilization, because for SRC a sufficient N supply can usually be generated from internal sources (mineralization of soil organic N, N_org_) or, at least in most parts of central Europe, even from the input of atmospheric N (Lamersdorf et al., 2010). On the other hand, site preparation for an SRC plantation (i.e., plowing, harrowing etc.) will stimulate mineralization and nitrification pulses of formerly accumulated N_org_. In consequence, an initial nitrate leaching pulse may occur under newly installed SRC plantations, and was already previously described (Goodlass et al., 2007; Lamersdorf et al., 2010; Schmidt-Walter and Lamersdorf, 2012).

For the present study, it is concluded that soil disturbances by site preparation and cultivation measures may have caused such initial nitrification pulses, especially under willow, because site conditions here are to some extent more favorable for such processes [higher total carbon contents due to enhanced percentage of clay and silt texture; for further details see Hartmann et al. (2014)]. Thus, the enhanced mean NNM under willow could also be taken as an indication for higher biological activities at this plot, and thus the risk for initial nitrification pulses after soil disturbances by cultivation measures. However, both SRC plantations already accumulate N in the range of almost 10–18 kg ha^−1^ a^−1^ in their initial growth phase. In addition, it is noticeable that the investigated poplar plantation is obviously more efficient in taking up nitrogen from the input (25%), compared to willow (14%). Vice versa, the willow plantation seems to be much more efficient in using the nitrogen taken up for wood production, as almost 200 kg DM of woody biomass were produced per 1 kg N, whereas poplar produced only 135 kg of DM with the same amount of consumed N. It can be summarized that the poplar and willow plantations investigated here show already in the initial growth stage obvious differences in their N-cycling and N-use efficiencies.

It has to be pointed out that the given N-budget calculations are only first estimates, i.e., the underlying measurements are just covering the first one or 2 years of the entire life of such SRC plantations. Furthermore, there might be clone specific effects, which were not further considered here. On the other hand, if such identified “traits” of certain SRC plantations with respect to the nitrogen cycling could be confirmed by further investigations, they could also be used for a target-oriented usage of SRC in the landscape. If for instance an N excess from the previous crop cultivation should be reduced, poplar as considered here (clone Max 1) seems to be more favorable compared to willow, due to its higher N-uptake efficiency. On the contrary, if less N is available on, e.g., naturally poor or long-time abandoned sites but high woody biomass production is still in focus, the here considered willow SRC (clone Tordis) seems to be more advisable.

Scenario results of the case study simulation illustrated that the vast majority of arable sites were not capable of providing economic return from SRC that outcompetes annual reference crops in all scenario cases. At first sight, this may lead to the fallacy that SRC is no option for farmers in the Göttingen district. But, despite of a relatively high price level of annual crops used in the case study scenario, MC revealed that around 19% of the arable sites provide conditions under which economic return grom SRC beats a reference crop rotation of wheat, oilseed rape, and barley under all circumstances. These sites are considered as “first choice” options since they fulfill the criterion of a low-risk alternative to annual crops and could be used to enlarge the regional renewable energy mix.

However, with a range between 0 and 180 €, the median minimum annuity-difference to the reference crop rotation is only +47 € ha^−1^, which raises the question if a risk-averse farmer will perceive this as an incentive to shift to SRC cropping.

Implementing SRC for a 20-year period results in a loss of flexibility and provides high initial investment costs for farmers. Thus, the farmer needs apart from spatial explicit information that allows for a thorough site selection, incentives to opt for perennial crops. Apart from much better adapted investment, subsidies toward lower thresholds of farmers implementation investments (Bemmann and Butler Manning, 2013) the most important prerequisite is confidence in reliable political boundary conditions, which in turn are the basis for the establishment of value- and supply chains which again support market penetration. This “vicious circle” or “chicken-and egg” problem is not only described for Germany (Bemmann et al., 2010; Thrän et al., 2011; Bemmann and Butler Manning, 2013) but was investigated for the UK as well (Sherrington et al., 2008; Sherrington and Moran, 2010). Overregulation for SRC compared to conventional crops in combination with only marginal consideration of ecological services provided by SRC in environmental payment schemes (Bemmann and Butler Manning, 2013) additionally hampers farmers to implement SRC.

However, as pointed out in the abovementioned studies, local and regional solutions could be an important step-stone to overcome some of the political and perception barriers. At this point, regional politics and actors play a crucial role. The Göttingen district, as many regions in Germany, is focusing on renewable energy strategies for climate protection and needs a mix of renewable energy carriers to fulfill the ambitious policy targets. Promoting the economic feasibility of proper SRC sites could provide an important incentive for farmers and a further renewable energy source helping to reach regional climate protection goals. This could be especially interesting on sites where ecosystem services can be combined with a positive economic return. In terms of erosion protection, case study results depicted that SRC could be a competitive option for around 3% (110 ha) of the arable sites in the study area. To breach the abovementioned “vicious circle,” regional policy must initiate more demonstration projects to illustrate if and how regional supply chains could work. For the Göttingen district, public buildings (e.g., schools) should be used as part of the climate protection agenda to establish local heating networks. Participatory approaches – as applied for the development of the integrated climate protection strategy (Landkreis Göttingen, 2013) – turned out to be very helpful to build social networks and to address the concerns of regional actors. When it comes to woody biomass supply for renewable energy generation, the authors deem the integration of ecological and economic considerations in a joint tool for scenario development and analysis as a promising way to develop a participatory decision support on a regional scale. Building on previous work by Busch (2012), BEAST combines ecological assessments with calculations of economic revenue as a basis for a participative regional dialog on sustainable land use and climate protection goals [see Busch and Thiele (2015) for further details]. Introduced on regional stakeholder workshops in 2014, BEAST will be applied in the regional LEADER process and case studies of the district energy agency as a first step toward regional implementation strategies of renewably energy supply from SRC in 2015.

## Conflict of Interest Statement

The authors declare that the research was conducted in the absence of any commercial or financial relationships that could be construed as a potential conflict of interest.
